# Text Neck-Induced Cervical Osteoarthritis in a Young Adult: A Case Report

**DOI:** 10.7759/cureus.113297

**Published:** 2026-07-24

**Authors:** Eric Chun-Pu Chu

**Affiliations:** 1 Chiropractic and Physiotherapy Centre, New York Medical Group, Hong Kong, CHN

**Keywords:** cervical osteoarthritis, cervical spine, chiropractic care, facet joint pain, forward head posture, mechanical traction, neck pain relief, posture correction, smartphone overuse, text neck

## Abstract

Text neck is a syndrome characterized by pain and discomfort in the neck and shoulders resulting from holding the head forward for extended periods while using smartphones or texting messages. With the widespread use of mobile devices, cervical osteoarthritis is becoming increasingly prevalent among young people. A 25-year-old college student presented with recurrent headaches and persistent neck and shoulder pain for five years. Her symptoms significantly worsened during the COVID-19 pandemic due to extended hours spent texting on her smartphone (approximately 8-10 hours per day) and participating in remote learning on her laptop. Cervical radiographs revealed hypertrophy of the facet joints and narrowing of intervertebral spaces, indicative of cervical osteoarthritis. Treatment consisted of multimodal chiropractic interventions such as thermal ultrasound therapy, mechanical vibration massage, spinal adjustments, and mechanical cervical traction. After 10 months of intervention, the patient reported reduced pain, improved spinal alignment, enhanced neck mobility, and increased intervertebral spacing. This case study highlights that a lack of awareness of head posture contributes to the development and progression of text neck, emphasizing the significance of early recognition and proper care.

## Introduction

Cervical osteoarthritis is a progressive degenerative joint disease characterized by cartilage breakdown, subchondral bone remodeling, and structural changes within the spinal joints. While historically considered an age-related condition affecting older adults, premature cervical osteoarthritis is increasingly recognized in young adults due to sustained postural stressors associated with prolonged digital device usage.

At each spinal level, the motion segment is formed by a tripod formed by two facet joints and an intervertebral disc. The stability of the motion segment and other components is impacted when one component undergoes a biomechanical change. The bilateral facet joints facilitate coupled intervertebral motions during axial rotation and bending, and they support more than 64% of the axial load of the cervical spine [1]. Facet articular apposition has been found to be influenced by axial neck stresses, which are modified by head posture and muscle activation [2]. Neck flexion compresses the cervical spine 1.6 times more than a neutral posture [3]. The degeneration of joint cartilage is the cause of facet joint osteoarthritis.

This case involves a college student diagnosed with text neck accompanied by cervical osteoarthritis. Radiographs showed reverse lordosis, facet joint hypertrophy, and narrowing of the intervertebral spaces. The patient reported pain relief and regained normal neck mobility after undergoing a multimodal chiropractic program aimed at improving cervical spine function. Spinal posture has a significant biomechanical impact on the distribution of stress in the facet joints. Joint degeneration can be mitigated by alleviating strain through ergonomic changes and properly addressing joint misalignments. To our knowledge, this case is among the few to provide objective radiographic evidence of increased intervertebral space and curvature restoration in a young adult with text neck-induced cervical osteoarthritis following non-surgical, multimodal conservative management. Highlighting these observable structural improvements underscores the potential for early conservative intervention to modify degenerative trajectories in tech-reliant populations.

## Case presentation

A 25-year-old female college student has been experiencing frequent headaches along with chronic neck and shoulder pain for the past five years. The pain typically lasts for hours, radiating from the sides of her neck to the occiput and extending down toward her shoulders. She described it as a dull ache with a moderate intensity, scoring 5-6 out of 10 on a numeric pain assessment scale. Occasionally, she experiences nausea during episodes where the headache becomes particularly severe. The patient noted that her symptoms worsened during the COVID-19 pandemic in Hong Kong, which forced her to rely extensively on laptops for online learning and messaging for an estimated 8-10 hours/day. The treatments provided by her family physician, which included prescribed analgesics, failed to relieve her discomfort. The persistent pain resulted in problems with sleep deprivation and hindered her ability to focus on her studies. In her quest for relief, she opted to seek chiropractic care to address her issues.

She denied any history of injuries or a family history of neurological disorders. She denied any prior history of physical neck trauma, head injuries, or a family history of neurological disorders. Further clinical inquiry confirmed no history of drug allergies, current or past hormonal therapy, or use of medications affecting calcium metabolism (such as long-term corticosteroids or anticonvulsants). Screening for red flags was negative, with no evidence, personal history, or systemic symptoms indicative of cervical tumors, neoplastic disease, or Pott's disease (spinal tuberculosis).

**Figure 1:**
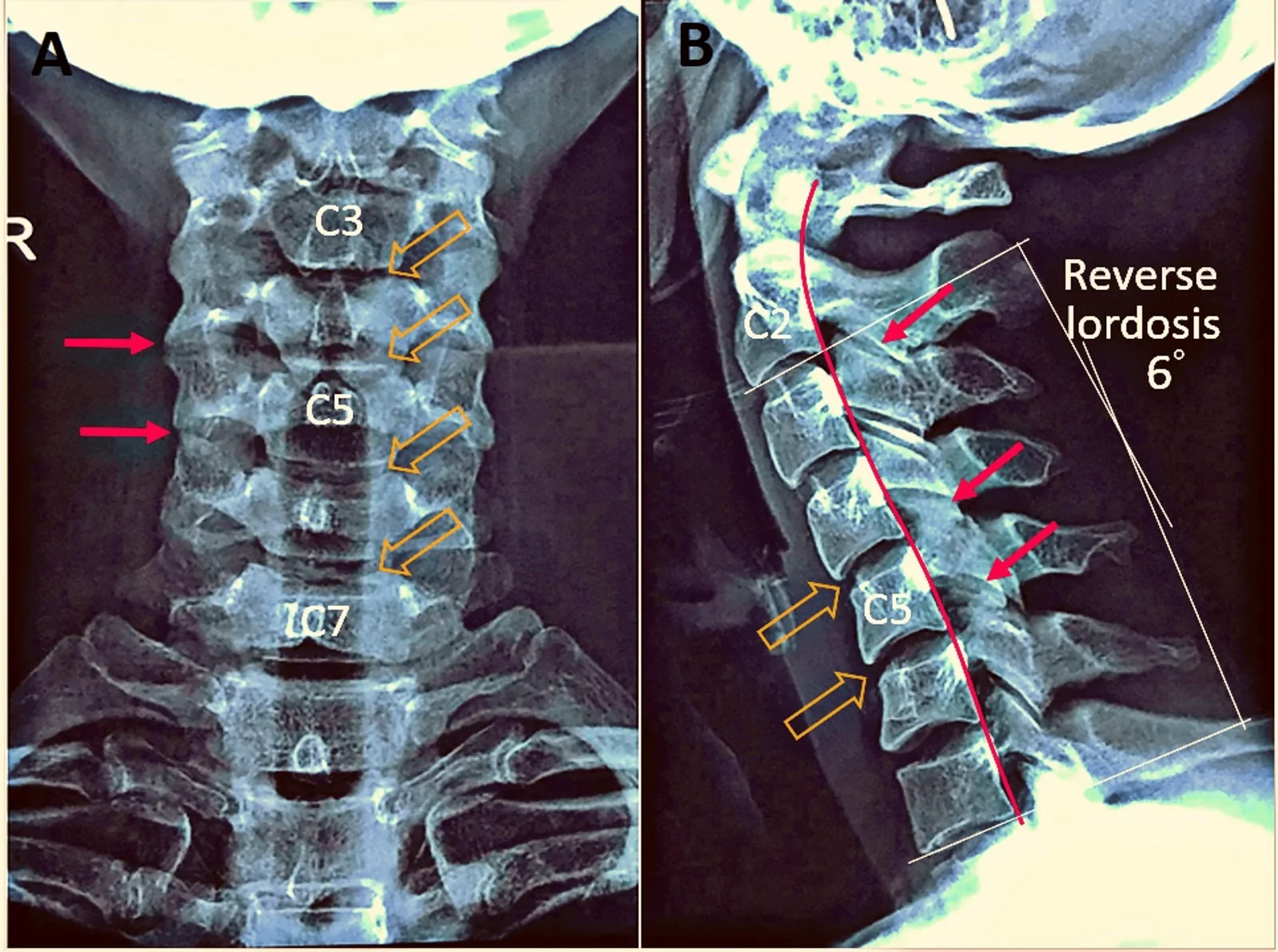
Cervical radiographs taken at the time of initial presentation Anteroposterior (A) and lateral neutral (B) views show cloudiness of the facet joints (red arrows), narrowing of intervertebral spaces (orange arrows), and reverse lordosis (Cobb C2-C7 6°). The red line highlights the posterior vertebral body margins. The radiographic findings are consistent with cervical spine osteoarthritis of the facet joint.

Upon initial examination, the patient presented with forward head posture, and her neck was straight with restricted extension, flexion, and rotation. Spurling's maneuver was negative, and the patient's nervous system was intact. Palpation demonstrated spasms and soreness in the bilateral cervical paraspinal, suboccipital, and upper trapezius muscles, as well as joint restriction in segments C3-C7. The laboratory results showed a normal blood count, creatinine, and electrolytes. A radiographic examination (Figure 1) revealed cloudiness of the facet joints (red arrows), narrowing of intervertebral spaces (white arrows), and reverse lordosis (Cobb C2-C7 6°). The results are in line with cervical spine osteoarthritis affecting the facet joints. Based on cervical radiographs and clinical symptoms, the patient was diagnosed with spondylitis of the cervical spine (ICD-10-CM M46.92).

Chiropractic treatment used thermal ultrasound therapy and a massage machine (G5® Massage Machine, General Physiotherapy, Inc., St. Charles, Missouri, United States) to improve blood flow and circulation in the neck and shoulders. Spinal manipulation was performed using the high-velocity, low-amplitude (HVLA) diversified technique. With the patient positioned in the supine position, the clinician applied precise, short-lever, posterior-to-anterior (P-A) and lateral-to-medial manipulative thrusts directed at the hypomobile C4-C6 segmental levels to restore normal facet joint motion and alleviate periarticular mechanical tension. Thermal ultrasound (1.0 MHz at 1.2 W/cm² for eight minutes) and percussive mechanical vibration massage were applied to the suboccipital and upper trapezius musculature prior to manipulation to achieve muscle relaxation. Mechanical traction (iTrac® Spine Remodeling System, Pivotal Health Solutions, Watertown, South Dakota, United States) was done to help restore movement in stiff neck and upper back areas. The patient had three chiropractic sessions per week for the first month. Following four weeks of consistent care, the patient demonstrated a 50% reduction in headache frequency and intensity, along with marked improvement in cervical range of motion. Her neck also moved much better. Then, she went weekly for three additional months. Four months following starting treatment, the patient reported no more headaches, neck, or shoulder pain. She could move her neck fully again. The patient continued chiropractic care twice a month for six months. The patient was given a home exercise program to strengthen her shoulder and neck. She was also given advice on how to improve her workspace, lifestyle tips, and a recommendation to limit her laptop and smartphone use to three hours.

**Figure 2:**
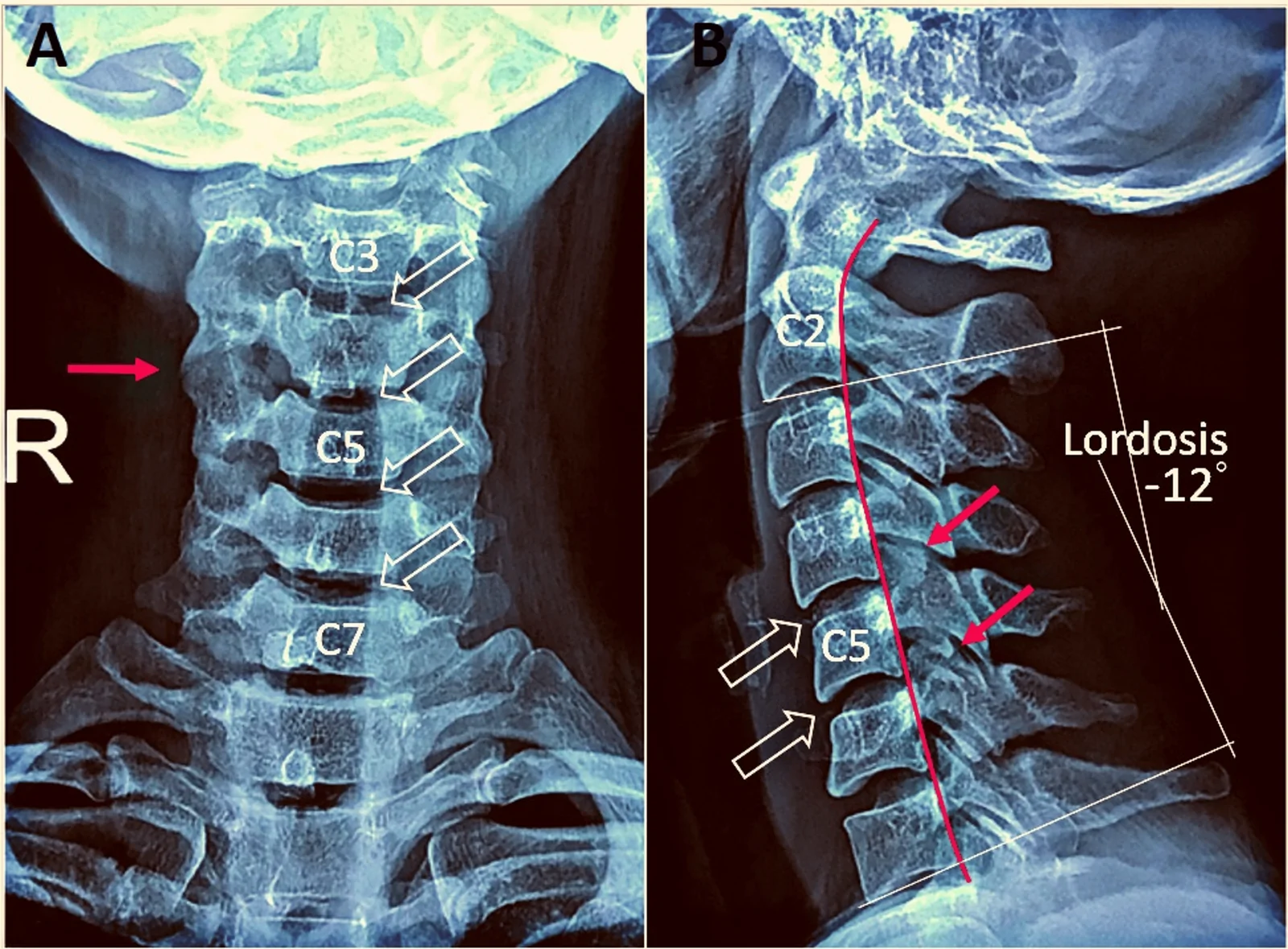
Repeat cervical radiographs taken 10 months after treatment initiation In comparison to the initial findings shown in Figure 1, there is noticeable improvement in the intervertebral spaces (white arrows) and facet joints (red arrows) in both anteroposterior (A) and lateral neutral (B) views, which display increased spacing. Additionally, the cervical curve shows restored lordosis with the C2-C7 Cobb angle measuring -12°.

In the follow-up radiograph (Figure 2) taken after 10 months of treatment, there was a restoration of cervical lordosis along with an increase in intervertebral spaces. The evaluation of pain intensity decreased from 6/10 to 1/10 on the rating scale. Subsequently, she continued to receive monthly maintenance therapy. No pathological or treatment-related adverse effects were noted.

## Discussion

This case reports a 25-year-old female college student with severe text neck-induced cervical osteoarthritis who experienced complete clinical symptom resolution, functional recovery, and notable radiographic restoration of cervical lordosis and intervertebral joint space following 10 months of conservative, multimodal chiropractic care.

Osteoarthritis serves as a general term for age-related deterioration that impacts the entire synovial joint, including the cartilage, subchondral bone, synovial tissue, and periarticular tissues [4,5]. With the exception of the atlantooccipital and atlantoaxial joints, facet joints (also known as zygapophyseal joints) are the only synovial joints in the spine. Facet joints participate in the same degenerative processes that contribute to the progression of osteoarthritis [6]. The intervertebral joints (disc joints) lack the synovial structures required to fulfill the criteria for osteoarthritis [7]. Osteoarthritis is generally accepted as a true disease, whereas degenerative disc disease is constantly viewed simply as an imaging description [8]. Accordingly, spondylosis (2022 ICD-10-CM Code M47) is primarily a term used to describe arthrosis or osteoarthritis of the spine and degeneration of the facet joints.

Cervical osteoarthritis or cervical spondylosis is no longer a condition that affects exclusively older adults; young adults are increasingly susceptible to early-onset degenerative joint disease due to chronic postural strain from modern digital device usage. Due to the growing popularity of media devices such as mobile devices and smartphones, the young population reporting chronic headache and neck pain and premature arthritis is increasing. Cervical degeneration and tissue wear are the most common causes of facet joint disorders in the spine. It is not unexpected that facet wear is attributed to prolonged neck flexion while looking down at a mobile screen. Continuous reinforcement of poor biomechanics, like excessive texting, is a constitutional factor facilitating cervical osteoarthritis. The bending of the neck forward will increase facet contact forces at adjacent segments [9], which contributes to the deterioration of the joints. Inflammation causes periarticular fibrosis and swelling of the joint capsules, resulting in stiffness and pain in the cervical spine [10]. Recognizing the association between radiographic findings and clinical complaints could offer useful insights for understanding the impact of the neck [6].

The capsule of the facet joints is richly innervated by mechanoreceptive and nociceptive afferents. Repeated stress of these afferents can trigger spinal sensitization through disrupted glutamatergic signaling, resulting in chronic pain and muscle weakness [11]. Unlike radiculopathy, facet arthropathy typically does not present with neurologic symptoms such as motor or sensory impairments or diminished reflexes. Pain originating from cervical facet joints of the lower neck often radiates to the shoulder girdle and interscapular region [6]. Degenerative changes in the facet joints may lead to neck stiffness, muscular spasms, and limitations in cervical mobility.

Currently, there are no specific treatments that can stop or reverse osteoarthritis or the degeneration of intervertebral discs. When conservative approaches do not succeed, interventional techniques such as facet joint injections, medial branch blocks, neurolysis, or surgical intervention are employed to alleviate pain [12]. In the scenario presented, the increased spacing of intervertebral and facet joints noted in the follow-up radiographs (Figure 2) may signify a favorable alteration associated with improved spinal function. Such advancements could stem from various effects of the manipulative procedures, including the correction of facet joint dysfunction [13], mobilization of restricted tissues, reinforcement of neck muscles, or relief of compressed tissues resulting from restricted mobility [14,15]. While cervical spinal adjustments are generally safe in appropriately screened patients, clinicians should exercise caution in those with conditions that may increase the risk of cervical artery dissection [16]. In this case, the patient denied any history of connective tissue disorders, and no adverse events were observed during treatment. 

Our findings align with previously documented cases demonstrating that severe postural strain from mobile devices can induce early degenerative changes and loss of lordosis in young populations [17]. Similar to reports where correction of reversed cervical lordosis relieved chronic radicular and structural symptoms in young adults, our case illustrates that mechanical unloading of the cervical column via traction and adjustments can halt symptom progression and encourage functional recovery [18,19]. Furthermore, while conventional medical management of cervical osteoarthritis frequently relies on oral analgesics or nonsteroidal anti-inflammatory drugs (NSAIDs), which provide temporary symptom suppression without correcting underlying sagittal malalignment, prior reports have demonstrated that conservative multimodal chiropractic care can effectively resolve refractory neck pain, headaches, and associated functional deficits [20].

However, several limitations inherent to this case report must be acknowledged. First, as a single-case study, the findings cannot be generalized to all young adults presenting with text neck or cervical osteoarthritis. Second, because a multimodal conservative care plan was utilized, combining thermal ultrasound, mechanical vibration, spinal manipulation, cervical traction, and ergonomic modifications, it is not possible to isolate the individual contribution or therapeutic efficacy of any single modality. Third, without a control group or long-term follow-up beyond 10 months, natural history, placebo effects, or long-term structural sustainability cannot be definitively established. Future prospective studies with larger sample sizes and long-term radiological monitoring are warranted to confirm these structural and functional outcomes.

## Conclusions

This case highlights three interconnected clinical takeaways for primary care and musculoskeletal providers. Firstly, prolonged, high-volume screen time ("text neck") can cause chronic postural strain that accelerates premature cervical osteoarthritis and facet joint degeneration in young adults. Secondly, text neck-induced cervical osteoarthritis should be considered in young patients presenting with chronic, recurrent headaches and neck pain that prove refractory to conventional oral analgesics. Lastly, non-invasive, multimodal chiropractic care, combining spinal adjustments, cervical traction, myofascial therapy, and ergonomic correction, can successfully achieve clinical symptom resolution while demonstrating objective radiographic improvements, including restored cervical alignment and increased intervertebral space. Early diagnostic recognition and structural biomechanical management offer an effective, non-pharmacological pathway to halt degenerative progression and restore spinal health in tech-reliant young populations.
